# Differences in HCV Seroprevalence, Clinical Features, and Treatment Outcomes between Female and Male Incarcerated Population: Results from a Matched Cohort Study

**DOI:** 10.3390/v15122414

**Published:** 2023-12-12

**Authors:** Vito Fiore, Andrea De Vito, Elena Rastrelli, Valentina Manca, Giuseppe De Matteis, Roberto Ranieri, Emanuele Pontali, Nicholas Geremia, Sandro Panese, Giulio Starnini, Giordano Madeddu, Sergio Babudieri

**Affiliations:** 1 Unit of Infectious Diseases, Department of Medicine, Surgery, and Pharmacy, University of Sassari, 07100 Sassari, Italy; andreadevitoaho@gmail.com (A.D.V.); valentimanca@gmail.com (V.M.); giordano@uniss.it (G.M.); babuder@uniss.it (S.B.); 2 PhD School in Biomedical Science, Biomedical Science Department, University of Sassari, 07100 Sassari, Italy; 3 Medicina Protetta-Unit of Infectious Diseases, Belcolle Hospital, 01100 Viterbo, Italy; elena.rastrelli@gmail.com (E.R.); hepan007@gmail.com (G.S.); 4 Health Protection for Adults and Youth Unit, Penitentiary Institute, 84124 Salerno, Italy; info@solentovacanze.it; 5 Penitentiary Infectious Diseases Unit, A.O. Santi Paolo e Carlo, University of Milan, 20122 Milan, Italy; roberto.ranieri@asst-santipaolocarlo.it; 6 Infectious Disease Unit, Galliera Hospital, 16128 Genoa, Italy; pontals@yahoo.com; 7 Unit of Infectious Diseases, Department of Clinical Medicine, Ospedale Dell’Angelo, 30174 Venice, Italy; nich.geremia@gmail.com (N.G.); sandro.panese@aulss3.veneto.it (S.P.); 8 Unit of Infectious Diseases, Department of Clinical Medicine, Ospedale Civile “S.S. Giovanni e Paolo”, 30122 Venice, Italy

**Keywords:** HCV, micro-elimination, gender medicine, penitentiary settings

## Abstract

Background: Women represent less than 5% of the incarcerated population in Italy, with very limited data on HCV infection. Higher HCV seroprevalence and active infection rates have been described among incarcerated females in available studies. Our aim is to compare the prevalence and cascade of care of HCV between male and female populations in Italian penitentiaries. Methods: We conducted a multicentre, retrospective study comparing HCV seroprevalence, active infections, treatment, and SVR rates between female (Group A) and male (Group B) populations in Italian prison settings. Results: No significant differences were found between the two groups regarding PWIDs (*p* = 0.16), nor in people living with HIV (*p* = 0.35) or HBV co-infection (*p* = 0.36). HCV seroprevalence was higher in Group A (*p* = 0.002). There was no statistically significant difference between the two groups regarding active infections (*p* = 0.41). Both groups showed a low level of fibrosis, and the dominant genotype was 3a. Almost all patients underwent antiviral treatment. All treated patients achieved SVR12. Conclusions: Our findings illuminate the importance of recognizing and addressing gender differences in HCV seroprevalence within penitentiary settings. Moving forward, addressing the unique needs of incarcerated females and optimizing HCV care for all incarcerated individuals are essential steps in the pursuit of achieving HCV micro-elimination goals.

## 1. Introduction

Globally, hepatitis C virus (HCV) infection remains a critical public health issue, with an estimated prevalence of 71 million individuals, many of whom are undiagnosed and untreated [1]. As a leading cause of liver-related morbidity and mortality, the virus presents unique challenges due to its asymptomatic nature in the early stages and potential for chronic disease progression [2]. It is characterized by its potential for chronic infection; HCV is a leading cause of liver cirrhosis and hepatocellular carcinoma (HCC) [2]. The World Health Organization (WHO) has targeted the elimination of HCV as a public health threat by 2030, a goal which necessitates focused efforts on high-prevalence populations, including those in correctional facilities. In particular, the HCV infection prevalence is higher among incarcerated individuals than in the general population [3]. Given their high-risk behaviors (e.g., people who inject drugs, PWIDs) and the possibility of HCV transmission upon release, incarcerated people represent one of the “special” subpopulations for HCV micro-elimination [4].

The advent of direct-acting antivirals (DAAs) has revolutionized the treatment landscape for HCV, consistently reducing the number of actively infected patients and consequently, the transmission of HCV [5,6]. DAAs have shown significant efficacy in reducing HCC recurrence and have substantially improved overall survival rates for HCV-infected patients [7,8]. Moreover, these therapies enhance liver function and lower all-cause mortality. The impact of DAAs extends beyond managing the virus itself; they profoundly improve patients’ overall quality of life. By effectively reducing the viral load and alleviating associated health complications, DAA therapy enables patients to experience significant improvements in both physical and mental well-being [9,10,11]. This holistic enhancement in quality of life, which includes better mental clarity, improved energy levels, and reduced HCV-related health distress, underscores the comprehensive benefits of DAAs in the treatment of HCV [12].

Over recent years, HCV prevalence in Italian penitentiary settings has drastically decreased from up to 38% in 2005, to approximately 10% in 2021. An increasing number of scientific reports are focusing on the feasibility and effectiveness of HCV therapy in prison. 

The role of gender influence in HCV epidemiology is complex and multifaceted. Women in correctional facilities often exhibit different risk profiles from their male counterparts, including higher rates of HCV seroprevalence [13]. This disparity is not solely a reflection of risk behaviors but also of social determinants of health that influence access to care and treatment outcomes. Understanding these gender-specific differences is paramount in tailoring interventions and optimizing the cascade of care for all individuals affected by HCV.

Moreover, the intersectionality of drug use, sex work, and incarceration creates a unique subset of the population at an elevated risk of HCV [14,15]. These factors necessitate a targeted approach to HCV screening and treatment, particularly for women who represent a smaller, yet significantly affected, demographic within prisons.

In reviewing the available national literature in this field, a notable limitation is the low proportion of included women (range 2.1–6.9%) [16]. As per the Italian Ministry of Justice official data, 58,987 people were incarcerated in Italian prisons on 30 September 2023. Of them, only 2498 were women [17].

Despite this, official data indicate that incarceration among women is more highly related to sex work and drug use, with these offenses often interrelated [17]. Additionally, a higher HCV seroprevalence and a higher rate of active infection have been described in incarcerated females compared to the general prison population [18,19,20]. Consequently, there is a growing recognition of the need for targeted interventions for the female population, and more data comparing female and male populations are needed. Understanding gender-specific differences in HCV seroprevalence and care among incarcerated individuals is essential for developing tailored intervention strategies and optimizing health outcomes. Furthermore, addressing these disparities is crucial in light of the global health goal of eliminating hepatitis C as a major public health threat by 2030 [21]. By exploring the differences in clinical features and the cascade of care between female and male incarcerated populations, we can better identify the unique needs and challenges faced by each group, thereby contributing to more equitable and effective healthcare solutions within the penitentiary system.

In light of these considerations, our study is poised to fill a critical gap in the literature by delineating the gender-specific prevalence and treatment outcomes of HCV in Italian correctional settings. By providing a comprehensive analysis of the HCV care continuum for both male and female incarcerated populations, we endeavor to contribute to the overarching goal of HCV elimination set forth by global health authorities.

## 2. Patients and Methods

We conducted a multicentre, retrospective study to compare HCV seroprevalence, active infections, treatment, and sustained virological response (SVR) rates between male and female populations in Italian prison settings. 

This study was specifically designed to provide insights into the gender differences in HCV prevalence and treatment outcomes within this unique environment.

Data extraction was meticulously carried out from the patients’ comprehensive medical records, ensuring accuracy and reliability in the representation of each patient’s health status and history of HCV treatment and progression.

### 2.1. Inclusion Criteria and Matching Parameters

In our study, participants were meticulously matched based on four key factors: age, primary HCV acquisition risk factor (with a focus on individuals who are people who inject drugs, PWIDs), and co-infections with HIV and hepatitis B virus (HBV). The study included only adult individuals (≥18 years old), ensuring that the data reflected the adult incarcerated population’s HCV status. We rigorously selected participants who had been previously evaluated by Infectious Diseases Specialists, ensuring a reliable medical history and accurate diagnosis for our analysis.

### 2.2. Sample Size and Statistical Analysis

The sample size was calculated based on the existing literature indicating a proportional HCV seroprevalence difference of 10.2% (10.3 vs. 20.5%) [19,20]. To achieve a statistical power of 80%, an alpha error of 5%, with an allocation ratio of 1:1, we determined that a total sample size of 390 patients (195 per group) was required for meaningful analysis. 

We assessed the normality of distribution using the Shapiro–Wilk test. Qualitative variables were summarized with absolute and relative (percentage) frequencies. The quantitative variables were described with the means ± standard deviation (SD) or medians and interquartile ranges (IQRs), according to the normality of their distribution. 

Continuous variables were compared with Student’s *t*-test or Mann–Whitney U test, based on their parametric and non-parametric distribution. Categorical variables were evaluated with Chi-squared or Fisher’s exact test, as appropriate. A two-tailed *p*-value less than 0.05 was considered statistically significant. All statistical computations were carried out with the statistical software STATA version 16 (StatsCorp, Lakeway, TX, USA).

### 2.3. Outcomes

Our primary outcome measures included the seroprevalence of HCV, determined by the detection of HCV antibodies among screened groups. HCV active infection was defined by the presence of detectable HCV RNA. 

The liver fibrosis was assessed using the FIB-4 index [22]. The treatment outcomes were categorized as the end of treatment (EOT), defined as completing the entire treatment schedule, and the SVR-12 defined as undetectable HCV RNA 12 weeks post-EOT. 

Virological failure was defined as detectable HCV RNA at EOT, while the breakthrough was defined as a new rise of viral load after initially achieving undetectability during treatment. Drop-outs were considered as unplanned interruptions after treatment initiation.

### 2.4. Treatments

Combination therapy was chosen following the indications of the Italian Association for the Study of the Liver (AISF) on the use of DAAs [23]. Our criteria for treatment initiation strictly adhered to the Italian Medicine Agency (AIFA) and Italian Association for the Study of Liver (AISF) recommendations. We ensured direct observation of treatment administration in prison settings to maintain adherence and accuracy in treatment delivery [23].

### 2.5. Ethical Issues

This study was conducted in accordance with the Declaration of Helsinki. All patients signed informed consent before participating, although data collection was anonymous. Our HCV micro-elimination protocol into the Italian Penitentiary System was approved by Istituto Superiore di Sanità, Roma, Italy—PRE BIO CE n. 38,762, req. 27 November 2018.

## 3. Results

### 3.1. Study Population

Our study included a total of 424 incarcerated individuals, comprising 196 females (Group A) and 228 males (Group B). The mean age was 44 ± 10.5 years for Group A and slightly lower for Group B at 41 ± 10.6 years, although this difference was not statistically significant (*p* = 0.33). In evaluating the risk factors and coinfection, no significant differences were found between the two groups in terms of the proportion of PWIDs (Group A vs. Group B = 38/196 (19.5%) vs. 57/228 (25%); *p* = 0.16), nor in people living with HIV (PLWH) (5 (2.6%) vs. 3 (1.3%); *p* = 0.35) or HBV co-infection (Group A vs. Group B = 3 (1.5%) vs. 7 (3%); *p* = 0.36).

However, the HCV seroprevalence was significantly higher in Group A than in Group B (Group A vs. Group B = 36/196 (18.4%) vs. 19/228 (8.3%)) with a *p*-value of 0.002.

The general characteristics of patients included in our study are reported in Table 1.

### 3.2. HCV Seroprevalence and Active Infections

Among those people who tested positive for HCV screening, there was no statistically significant difference between the two groups regarding active infections (Group A vs. Group B = 23/36 (63.8%) vs. 10/19 (52.6%); *p* = 0.41). The predominant HCV genotype identified was genotype 3a observed in 56.5% of Group A (13/23) and 70% (7/10) of Group B, but this difference was not statistically significant (*p* = 0.26). Concerning liver fibrosis, assessed using the FIB-4 value, the majority of patients in both groups showed a low level of fibrosis with no significant differences observed (Group A vs. Group B = 18/23 (78.3%) vs. 6/10 (60%); *p* = 0.4). The clinical features of patients who tested positive for HCV RNA are reported in Table 2.

### 3.3. HCV Treatment Cascade of Care

**Figure 1:**
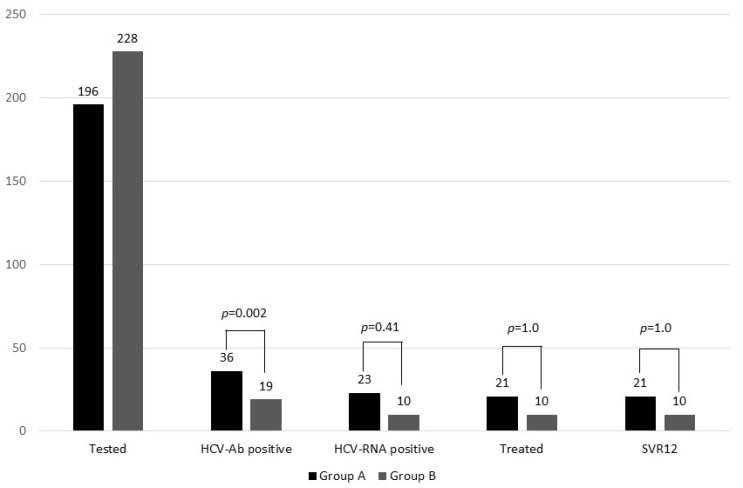
Differences in HCV seroprevalence, active infections, treatments, and outcomes between two groups of incarcerated people included in our study. Group A, incarcerated women; Group B, incarcerated men.

In terms of antiviral treatment, two individuals in Group A did not start treatment due to their release before prescriptions could be administered. In contrast, all patients in Group B started the treatment. Despite this, there were no statistically significant differences in treatment initiation between these two groups (Group A vs. Group B = 21/23 (91.3%) vs. 10/10 (100%); *p* = 1.0). The treatment choice was glecaprevir/pibrentasvir in 11/21 (52.4%) treated patients in Group A and 7/10 (70%) in Group B. All other patients received sofosbuvir/velpatasvor. Remarkably, all treated patients who began treatment in both groups successfully achieved EOT and SVR 12, with no instances of virological breakthroughs observed. No patients experienced adverse reactions during treatment. The HCV cascade of care among patients included in our study has been reported in Figure 1, highlighting the differences in HCV seroprevalence, active infections, treatments, and outcomes between the two groups.

## 4. Discussion

Our study, centered on comparing the prevalence and the cascade of care of HCV in male and female populations in Italian penitentiary settings, has revealed several critical findings with far-reaching implications. Notably, we observed a higher HCV seroprevalence in the female incarcerated population compared to their male counterparts. This trend is concordant with the existing literature, which highlights a close association between incarceration in women and high-risk behaviors, such as sex work and drug use. This significant finding underscores the pressing need for gender-specific interventions and preventive measures, aimed at addressing the unique risk factors and vulnerabilities faced by women in penitentiary settings.

Interestingly, despite the differences in seroprevalence, our study’s findings regarding the absence of significant disparities in active infections, genotype distribution, fibrosis levels, and treatment outcomes between genders are particularly noteworthy. This suggests that the effectiveness of current treatment protocols within the prison system is commendable, indicating that the unique challenges of the prison environment do not inherently disadvantage one gender over another regarding HCV treatment outcomes.

In the vast landscape of existing literature on HCV, our study presents intriguing contrasts and parallels, especially when considering gender-based differences in HCV seroprevalence. Our observation of a notably higher HCV seroprevalence in females (18.4%) as compared to males (8.3%) within Italian penitentiary settings starkly contrasts with findings from different geographical locations. For instance, the study conducted by Marco Antonio Moreira Puga et al. revealed a significantly lower prevalence in females (0.6%) compared to males (2.7%) in Brazilian prisons [24]. This discrepancy not only highlights the variability in HCV prevalence across different settings but also underscores the influence of socio-cultural and environmental factors in disease transmission and prevalence. Similarly, the study by Abdel-Gawad et al., which demonstrated a higher HCV RNA positivity in males than females among the adult population in Egypt, further supports the notion of a geographically influenced and gender-specific nature of HCV prevalence [25]. Such geographical disparities in prevalence rates suggest that HCV transmission dynamics and risk factors are deeply intertwined with local cultural, social, and healthcare practices. 

The variability in risk factors and incidence rates across different populations, as exemplified by the Central Brazil study, underscores the critical importance of local contexts in shaping HCV transmission dynamics. It also highlights the need for developing intervention strategies, which are finely tuned to these local nuances. In this regard, our study contributes significantly to the existing body of knowledge by offering a detailed examination of HCV prevalence within Italian penitentiary settings, a context which has been less explored in the available literature.

On a broader scale, Dana Busschots et al.’s meta-analysis, which highlighted the extensive variability in HCV prevalence globally, places our study in a global context [26]. It brings to light Italy’s position as one of the countries grappling with high HCV/HIV co-infection rates. Our findings, therefore, contribute to a more nuanced understanding of HCV prevalence in Italy, underscoring the need for region-specific research and interventions tailored to local needs. This is particularly crucial in aligning with the World Health Organization’s ambitious goal of eliminating HCV by 2030 [21]. Additionally, our study’s detailed insights into the clinical features and treatment cascade of care for HCV-positive patients shed light on the effectiveness of current treatment protocols and help identify areas for improvement to ensure equitable healthcare within penitentiary systems. It emphasizes the importance of continuous evaluation and adaptation of healthcare strategies to address the unique challenges faced by incarcerated populations, particularly in the context of infectious diseases like HCV.

The observation of uniform success in HCV treatment outcomes across genders in our study holds profound implications for public health, particularly within the unique context of prison settings. This uniformity not only underscores but also reinforces the potential of these settings as effective venues for targeted HCV micro-elimination efforts. Prisons, often housing marginalized populations who might otherwise lack access to essential healthcare services, present a unique opportunity for public health interventions. By focusing on these healthcare providers can deliver targeted treatments to a segment of the population that is frequently overlooked in broader public health campaigns. This approach is not only crucial for improving the health outcomes of incarcerated individuals but also has the potential to contribute significantly to the wider public health goal of eliminating HCV.

Furthermore, the distinct epidemiological pattern among incarcerated females in our study, points to the need for a more tailored approach to addressing HCV within the prison population. The higher seroprevalence of HCV among incarcerated females highlights the necessity of developing and implementing tailored screening, prevention, and treatment strategies that are specifically designed to meet the unique needs and risk factors associated with these subgroups. Tailored interventions, which take into account the specific challenges and health risks faced by incarcerated females, could play a pivotal role in effectively reducing HCV transmission and improving overall health outcomes within this subpopulation.

An additional critical aspect is represented by the challenges associated with the continuity of care for incarcerated individuals undergoing antiviral treatment. Specifically, our results indicated that two individuals in Group A were unable to commence their antiviral treatment due to their release from the penitentiary system before prescriptions could be administered. This situation highlights a significant gap in the healthcare system within correctional facilities, particularly regarding the management of infectious diseases like HCV. The issue of releasing people who are in the midst of or about to start medical treatment poses a complex challenge. It disrupts the continuity of care, potentially leading to the loss of follow-up and the incomplete treatment of HCV, thereby affecting the individual’s health outcomes and increasing the risk of ongoing HCV transmission in the community. This disruption is particularly problematic in the case of HCV, where the effectiveness of treatment is highly dependent on adherence and completion of the prescribed regimen.

Moreover, the loss of follow-up due to people’s release underscores the need for integrated healthcare systems, that extend beyond the confines of correctional facilities. There is a pressing need for strategies that ensure a seamless transition of care from the prison healthcare system to community healthcare services. This transition is essential not only for the successful completion of HCV treatment but also for maintaining the broader public health benefits of reducing HCV transmission within the community. Addressing this gap requires the collaboration of prison health services, community healthcare providers, and policy-makers to develop effective mechanisms that facilitate the continuity of care. Such mechanisms might include the establishment of transitional healthcare programs, enhanced communication between prison and community healthcare systems, and the implementation of follow-up protocols that are initiated prior to a person’s release. These efforts are crucial in ensuring that individuals released from correctional facilities continue to receive the necessary healthcare support, particularly in the management of conditions like HCV. In response to this, several projects focused on retention in care with infectious diseases, and methadone clinics have proven effective in preventing the loss to follow-up after release in Italy [19,27]. These initiatives serve as a valuable model, demonstrating successful strategies for managing HCV and ensuring treatment completion. Such models are vital for global implementation, as they offer a structured approach to maintaining healthcare continuity for individuals with HCV, especially those with a history of drug addiction, post-release from correctional facilities. We encourage the adoption of similar strategies worldwide to enhance individual health outcomes and support broader public health efforts in facing HCV.

Additionally, our study inadvertently shed light on a significant issue regarding hepatitis B virus (HBV) vaccination among the incarcerated population. We found that a considerable number of the individuals included in our study were not vaccinated against HBV. This finding is particularly concerning given the high turnover of individuals in correctional facilities, which can pose significant challenges to the implementation of comprehensive vaccination campaigns. These challenges are often more pronounced among specific groups, such as older people and those from foreign countries, who may face additional barriers to accessing healthcare services. Addressing the issue of HBV vaccination in correctional facilities is crucial, not only for the health of the individuals within these facilities but also for the broader public health implications. Developing effective strategies to increase HBV vaccination coverage among incarcerated populations should be a priority for healthcare providers and policymakers working in the context of Italian correctional facilities. This approach would not only help safeguard the health of incarcerated people but also contribute to the broader public health efforts in controlling HBV transmission. 

It is imperative to acknowledge that our study does have certain limitations, primarily stemming from its retrospective nature and reliance on medical records. This methodological approach inherently carries the risk of biases and the presence of unmeasured confounders, which could influence the study’s findings. Additionally, the data extraction from medical records might not capture the complete spectrum of clinical and socio-behavioral aspects associated with HCV infection, potentially overlooking factors that might play a role in understanding the disease dynamics within the incarcerated population. The specificity of the context and population under study in our research also necessitates caution in generalizing the findings. Future research endeavors should aim to delve deeper into the underlying reasons for the observed higher seroprevalence of HCV among incarcerated females. It is crucial to scrutinize the interplay between specific risk behaviors, such as drug use and sexual practices, and broader social determinants, including access to healthcare, social support systems, and the impact of incarceration on health. Understanding these dynamics is key to developing more effective strategies for HCV prevention and treatment within this population.

There is a marked need for studies that examine the efficacy of gender-tailored interventions and explore barriers to HCV care among incarcerated populations to further inform and refine policy and practice.

## 5. Conclusions

Our study highlights the urgent need for gender-specific strategies in managing HCV within Italian penitentiary settings, revealing notable differences in HCV seroprevalence between incarcerated males and females. This disparity calls for tailored healthcare interventions, particularly for the more affected female population. Recognizing prisons as key venues for HCV intervention, our findings emphasize the importance of developing and implementing focused treatment programs in these settings. Addressing these gender disparities is not only vital for improving individual health outcomes but also crucial for advancing towards HCV micro-elimination goals in Italy. Future efforts should concentrate on integrating these insights into healthcare policies, ensuring equitable and effective HCV management across both genders in penitentiary environments. By doing so, we move closer to achieving broader public health objectives, including the global aim of eliminating HCV.

## Figures and Tables

**Table 1:** General characteristics of two groups of incarcerated people included in our case–control study.

Variable	Results		*p*-Value
	Group A (*n* = 196)	Group B (*n* = 228)	
Age, mean ± SD	44 ± 10.5	41 ± 10.6	0.33 *
Italian nationality, *n* (%)	127 (64.8)	162 (71.1%)	0.17 **
PWID, *n* (%)	38 (19.5)	57 (25)	0.16 **
On OST	14/38 (36.8)	33/57 (57.9)	0.44 **
PLWH (*n*, %)	5 (2.6)	3 (1.3)	0.35 **
HBV, *n* (%)			
HBsAg positive	3 (1.5)	7 (3)	
Anti-HBsAg positive	95 (48.5)	111 (48.7)	0.36 ***
Vaccined	8/95 (8.4)	13/111 (11.7)	0.96 **
Past infection	87/95 (91.6)	98 (88.2)	0.43 **
OBI	9 (4.6)	-	0.77 **
HCV-Ab positive, *n* (%)	36 (18.4)	19 (8.3)	0.002 **
HCV RNA positive	23/36 (63.8)	10/19 (52.6)	0.41 **

* Student’s *t*-test; ** Chi-squared test; *** Fisher’s exact test. Group A, female incarcerated people; Group B, male incarcerated people. SD, standard deviation; PWID, people who injected drugs; OST, opioid substitution therapy; PLWH, people living with HIV; OBI, occult HBV infection.

**Table 2:** Clinical characteristics of two groups of incarcerated people who tested positive for HCV RNA included in our case–control study.

Variable	Results		*p*-Value
	Group A (*n* = 23)	Group B (*n* = 10)	
HCV RNA, median (Q1-3)	566,000 (41,200–1,700,000)	481,381 (128,060–1,020,362.3)	0.89 *
HCV genotype, *n* (%) 1a 1b 2 3a 4	4 (17.4) 2 (8.7) 1 (4.4) 13 (56.5) 3 (13)	1 (10) 1 (10) - 7 (70) 1 (10)	1.0 ** 1.0 ** 1.0 ** 0.26 ** 1.0 **
FIB-4 value, *n* (%) Low (<1.45) Intermediate (1.45–3.25) High (>3.25)	18 (78.3) 3 (13) 2 (8.7)	6 (60) 2 (20) 2 (20)	0.4 ** 0.63 ** 0.57 **
HBV co-infection	2 (8.7)	-	-

* Mann–Whitney U test; ** Fisher’s exact test.

## Data Availability

Data will be available upon specific request.
